# Molecular pathogenesis of metabolic dysfunction‐associated steatotic liver disease, steatohepatitis, hepatic fibrosis and liver cirrhosis

**DOI:** 10.1111/jcmm.18491

**Published:** 2024-06-18

**Authors:** Takashi Saito, Mutsumi Tsuchishima, Mikihiro Tsutsumi, Joseph George

**Affiliations:** ^1^ Department of Hepatology Kanazawa Medical University Uchinada Ishikawa Japan; ^2^ Center for Regenerative Medicine Kanazawa Medical University Hospital Uchinada Ishikawa Japan

**Keywords:** hepatic fibrosis, liver cirrhosis, MASH, MASLD, steatohepatitis, steatosis

## Abstract

Metabolic dysfunction‐associated steatotic liver disease (MASLD) is characterized by intense deposition of fat globules in the hepatic parenchyma that could potentially progress to liver cirrhosis and hepatocellular carcinoma. Here, we evaluated a rat model to study the molecular pathogenesis of the spectrum of MASLD and to screen therapeutic agents. SHRSP5/Dmcr rats were fed a high‐fat and cholesterol (HFC) diet for a period of 12 weeks and evaluated for the development of steatosis (MASLD), steatohepatitis, fibrosis and cirrhosis. A group of animals were sacrificed at the end of the 4th, 6th, 8th and 12th weeks from the beginning of the experiment, along with the control rats that received normal diet. Blood and liver samples were collected for biochemical and histopathological evaluations. Immunohistochemical staining was performed for α‐SMA and Collagen Type I. Histopathological examinations demonstrated steatosis at the 4th week, steatohepatitis with progressive fibrosis at the 6th week, advanced fibrosis with bridging at the 8th week and cirrhosis at the 12th week. Biochemical markers and staining for α‐SMA and Collagen Type I demonstrated the progression of steatosis to steatohepatitis, hepatic fibrosis and liver cirrhosis in a stepwise manner. Control animals fed a normal diet did not show any biochemical or histopathological alterations. The results of the present study clearly demonstrated that the HFC diet‐induced model of steatosis, steatohepatitis, hepatic fibrosis and cirrhosis is a feasible, quick and appropriate animal model to study the molecular pathogenesis of the spectrum of MASLD and to screen potent therapeutic agents.

## 1 INTRODUCTION

Metabolic dysfunction‐associated steatotic liver disease (MASLD), which is the new name for non‐alcoholic fatty liver disease (NAFLD), is a clinicopathologic condition mostly associated with metabolic syndrome and a major health problem as well as an economic burden in developed as well as developing nations.^1^, ^2^ MASLD could lead to a series of problems ranging from hepatic inflammation to steatosis, steatohepatitis, advanced fibrosis and liver cirrhosis.^3^, ^4^ Based on lifestyle and aetiology, some cases of MASLD could progress to hepatocellular carcinoma, leading to death.^5^, ^6^ Metabolic syndrome and the associated insulin resistance, oxidative stress, lipid peroxidation and proinflammatory cytokines have significant contributions to the pathogenesis and progression of MASLD.^7^, ^8^ This is very true in most developed nations with a sedentary lifestyle, where the imbalance between caloric intake and caloric output is steadily increasing.^9^, ^10^ The advanced stage of MASLD with liver cirrhosis is non‐reversible, and liver transplantation is the only option of treatment.^11^ However, due to the shortage of organs available for transplant and the associated complications and rejection after transplantation, alternative treatment methods are a requisite.^12^ Therefore, we need a suitable animal model that is appropriate to study the molecular mechanisms associated with the pathogenesis of MASLD and to screen effective therapeutic agents that could arrest the progression of the disease.

The stroke‐prone spontaneously hypertensive rat/Disease models co‐operative research (SHRSP5/Dmcr), formerly called the arteriolipidosis‐prone rat, is the 5th substrain of SHRSP registered at the National BioResource Center, Tsukuba, Japan. In order to develop a MASLD model of quickly accumulating arterial fat deposition (arteriosclerosis) in response to a high‐fat and high‐cholesterol (HFC) diet, the new substrain has been produced by selective brother–sister inbreeding of SHRSP with stronger hypercholesterolemic responses (about 600–900 mg/dL for females and 300–600 mg/dL for males) to an HFC diet and water ad libitum for 1 week.^13^ This animal model was evaluated before and demonstrated that feeding with HFC diet can develop hepatic lesions similar to those in human metabolic dysfunction‐associated steatohepatitis (MASH) pathology.^14^ In the present study, we developed an appropriate animal model for the spectrum of MASLD ranging from simple steatosis to steatohepatitis (MASH), advanced hepatic fibrosis and liver cirrhosis in a stepwise manner after feeding SHRSP5/Dmcr rats with HFC diet for 12 weeks.

## 2 MATERIALS AND METHODS

### 2.1 Animals

Around 8‐week‐old SHRSP5/Dmcr male rats (body weight: 150 ± 8 g) were procured from the Disease Models Co‐operative Research Association (DMCRA), Kyoto, Japan. The animal experiments were carried out as per the Guide for the Care and Use of Laboratory Animals published by the US National Institutes of Health (NIH Publication No. 86‐23, revised 1996). The protocol was also approved by the Animal Care and Research Committee of Kanazawa Medical University on the Ethics of Animal Experiments (# 2018‐29). The animals were housed in stainless steel wire mesh cages in air‐conditioned rooms with a relative humidity of 50 ± 10% and frequent air changes. All the animals had automatic 12 h light and dark cycles and food and water available ad libitum.

### 2.2 Experimental procedures

The animal experiments were carried out in the animal house and the adjacent laboratories at Kanazawa Medical University, Japan. A total of 40 rats were used in the study. They were divided into eight groups of five rats each, and four groups were fed a high‐fat and high‐cholesterol (HFC) diet (Funabashi Farm, Chiba, Japan). The remaining four groups were fed a normal diet (Funabashi Farm, Chiba, Japan) and treated as controls. The nutrient composition of both the normal diet and the HFC diet is presented in Table 1. A group of five rats each from the normal and HFC diet were sacrificed at the 4th, 6th, 8th and 12th weeks from the beginning of the experiment. All the animals were anaesthetised with isoflurane before euthanasia, and blood was collected from the right jugular vein. The animals were euthanized under deep anaesthesia using a rodent guillotine, and excess blood was allowed to drain out. About 3 mm of thick liver tissue was cut from the median lobe and instantly fixed in 10% phosphate‐buffered formalin for histopathological studies. Another portion of the liver was frozen at −80°C for biochemical analyses. Animal body weight and liver wet weight were measured during the entire course of the study, and the liver wet weight to body weight ratio was calculated.

**TABLE 1:** Nutrient composition of normal diet and HFC diet used in the present study. The total energy was 3.29 kcal/g in normal diet and 5.72 kcal/g in HFC diet.

	Normal diet	HFC diet
Diet composition %		
Normal diet	100	68
Palm oil	–	25
Cholesterol	–	5
Cholic acid	–	2
Ingredients %		
Crude protein	18.8	14.1
Crude lipid	3.9	35.3
Crude fibre	6.6	2.2
Crude ash	6.9	3.4
Moisture	9.2	5.4
Carbohydrate	54.6	39.6

### 2.3 Measurement of aspartate aminotransferase and alanine aminotransferase in serum

Blood was allowed to clot at 37°C for 3–5 h, and serum was separated after centrifugation at 3000 rpm in a clinical centrifuge. Aspartate aminotransferase (AST) and alanine aminotransferase (ALT) present in the serum were measured using an auto‐analyzer (Fuji Drychem NX700, FujiFilm, Minato‐ku, Tokyo, Japan) for animal samples. AST and ALT values are presented as International Units per litre (IU/litre) of serum.

### 2.4 Determination of malondialdehyde and glutathione in the liver tissue

Malondialdehyde (MDA) is a characteristic marker of lipid peroxidation and an indicator of elevated cellular oxidative stress.^15^ Hepatic MDA content in the liver tissue was measured based on a fluorometric technique employing a MDA assay kit (Cat# NWK‐MDA01, Northwest Life Science, Vancouver, WA, USA). About 100 mg of frozen liver tissue was homogenized in 1 mL of ice‐cold 50 mM Tris–HCl buffer (pH 8) containing 150 mM NaCl, 1 mM EDTA, and 1% Triton X‐100. Then 50 μL of the homogenate was treated with 150 μL of 5% 5‐sulfosalicylic acid solution and vortexed well. It was allowed to stand on ice for 10 min and centrifuged at 12,000 × *g* for 10 min at 4°C. Hepatic MDA content was determined in the supernatant, diluted 1:1 with distilled water. Hepatic MDA content is presented as nmoles/g of wet liver tissue.

Reduced glutathione (GSH), a tripeptide (γ‐glutamyl‐cysteinyl‐glycine), is the major free thiol in the biological system and the most potent scavenger of superoxide and free radicals. GSH content was measured in the liver tissue using 50 μL of the above supernatant (diluted to 100 μL with distilled water) and employing a glutathione assay kit (Cat# CS0260, Millipore‐Sigma, St. Louis, USA). Hepatic GSH content is presented as μmoles/g of wet liver tissue.

### 2.5 Histopathological evaluation of the liver tissue

The histopathological changes during the pathogenesis of MASH and the development of hepatic fibrosis and early cirrhosis were evaluated in the liver tissue of HFC diet‐treated animals during the course of the study. The formalin‐fixed tissue slices were processed in an automatic tissue processor optimized for liver tissue, embedded in paraffin blocks and cut into sections of 5 μm thickness. The sections were stained with haematoxylin and eosin (H&E) to examine histopathological changes and Azan trichrome to study the extent of collagen deposition in the hepatic parenchyma. The stained sections were examined with an Olympus BX53 microscope attached with a DP 71 digital camera (Olympus Corporation, Tokyo, Japan) and photographed. The histopathological evaluation and grading were performed by a pathologist who was unaware of the time points of the study and the course of the treatment. The changes in pathological features during HFC diet administration were scored as per the method of Bedossa et al.^16^ and are reported as SAF score (steatosis, activity [comprises hepatocyte ballooning and lobular inflammation], and fibrosis). The steatosis score (S) was assessed as the quantity of large or medium‐sized lipid droplets, from 0 to 3 (S0: <5%; S1: 5%–33%, mild; S2: 34%–66%, moderate; S3: >67%, marked). Activity grade (A) was scored from 0 to 3 as A0 (no activity), A1 (mild activity), A2 (moderate activity) and A3 (severe activity). The stages of fibrosis (F) were assessed as follows; F0 (no fibrosis), F1 (portal and periportal fibrosis), F2 (bridging fibrosis) and F3 (advanced fibrosis and early cirrhosis). The average grade was calculated after examining 10 lobules on each liver section.

### 2.6 Immunohistochemical staining for α‐smooth muscle actin and Collagen Type I

The activation and transformation of round resting hepatic stellate cells into elongated myofibroblast‐like cells with the expression of α‐smooth muscle actin (α‐SMA) is considered a characteristic feature of the initiation of hepatic fibrosis.^17^ The prominent deposition of newly formed Type I collagen fibres in the hepatic parenchyma is a distinctive feature of hepatic fibrosis and early cirrhosis.^18^ In order to obtain explicit information about the pathogenesis of hepatic fibrosis during the progression of MASLD with the HFC diet, we performed immunohistochemical staining for α‐SMA and Collagen Type I in formalin‐fixed paraffin liver sections. The deparaffinized and hydrated liver sections were blocked with a protein block (Cat# ab64226) for 30 min at room temperature to prevent non‐specific binding. After washing thrice in cold phosphate‐buffered saline (PBS), the liver sections were treated with primary antibodies against α‐SMA (Cat# ab5694) and Collagen Type I (Cat# ab34710) (Abcam, Chuo‐ku, Tokyo, Japan) and incubated in a moisturized slide chamber (Evergreen Scientific, Los Angeles, CA, USA) at 4°C overnight. The sections were then washed three–five times in PBS and incubated with broad‐spectrum biotinylated secondary antibody for 2 h at room temperature. The slides were washed again with PBS, treated with streptavidin‐peroxidase conjugate and incubated for another 1 h. The final stain was developed after treating the sections with 3% 3‐amino‐9‐ethylcarbazole (AEC) in N, N‐dimethylformamide for 5–10 min. The stained sections were washed and counterstained with Mayer's haematoxylin for 2 min, mounted using aqueous‐based mounting medium and dried on air. The slides were examined under a microscope (Olympus BX53, Tokyo, Japan) fixed with a digital camera (Olympus DP71) and photographed.

### 2.7 Data analysis and statistics

Arithmetic mean and standard deviation (SD) were calculated for all the data and presented as Mean ± SD. The data were analysed and compared either by using analysis of variance (ANOVA) or Student *t‐*test, whichever was appropriate. A value of *p* < 0.05 was considered statistically significant.

## 3 RESULTS

### 3.1 Animal body weight and liver wet weight

**FIGURE 1:**
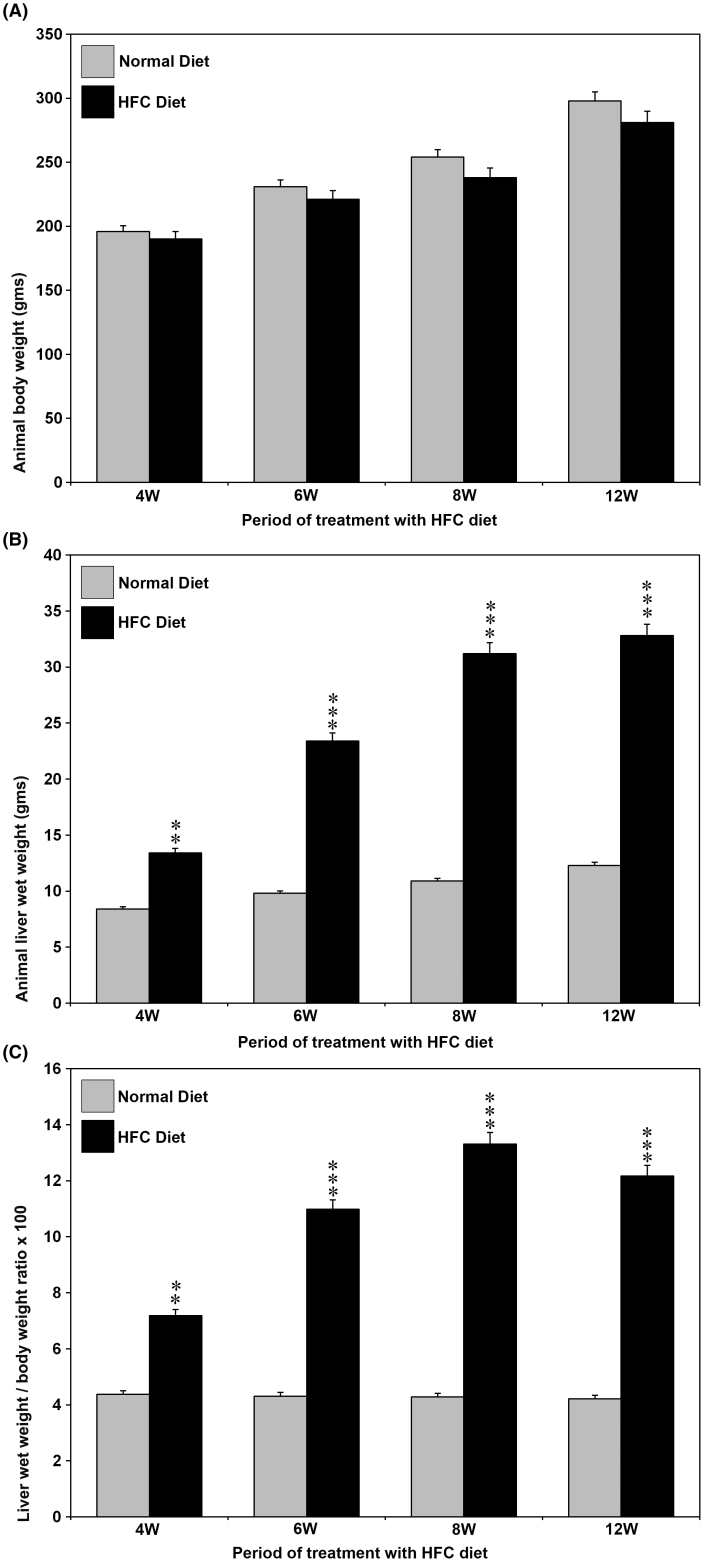
Body weight and liver wet weight as well as liver wet weight to body weight ratio of the animals treated with HFC diet. (A) Body weight. There was no significant difference between the mean body weight of control and HFC‐diet fed animals. (B) Liver wet weight. There was a significant increase in the liver wet weight of the animals fed the HFC diet. (C) Liver wet weight to body weight ratio (×100). The liver weight to body weight ratio was significantly increased in the HFC diet group in all the weeks studied. **p < 0.01 and ***p < 0.001 compared to the respective controls by ANOVA.

The animal body weight and liver wet weight, as well as the liver wet weight to body weight ratio during the course of HFC diet administration in SHRSP5/Dmcr rats, are presented in Figure 1. There was no difference in body weight between control and HFC diet‐fed animals during the course of the study (Figure 1A). The liver weight was significantly increased in the HFC diet group at all time points studied compared with the animals fed a normal diet (Figure 1B). The liver wet weight to body weight ratio was significantly increased in the HFC diet group compared with controls during the entire course of the study (Figure 1C).

### 3.2 Serum levels of AST and ALT during the pathogenesis of MASH and hepatic fibrosis

**FIGURE 2:**
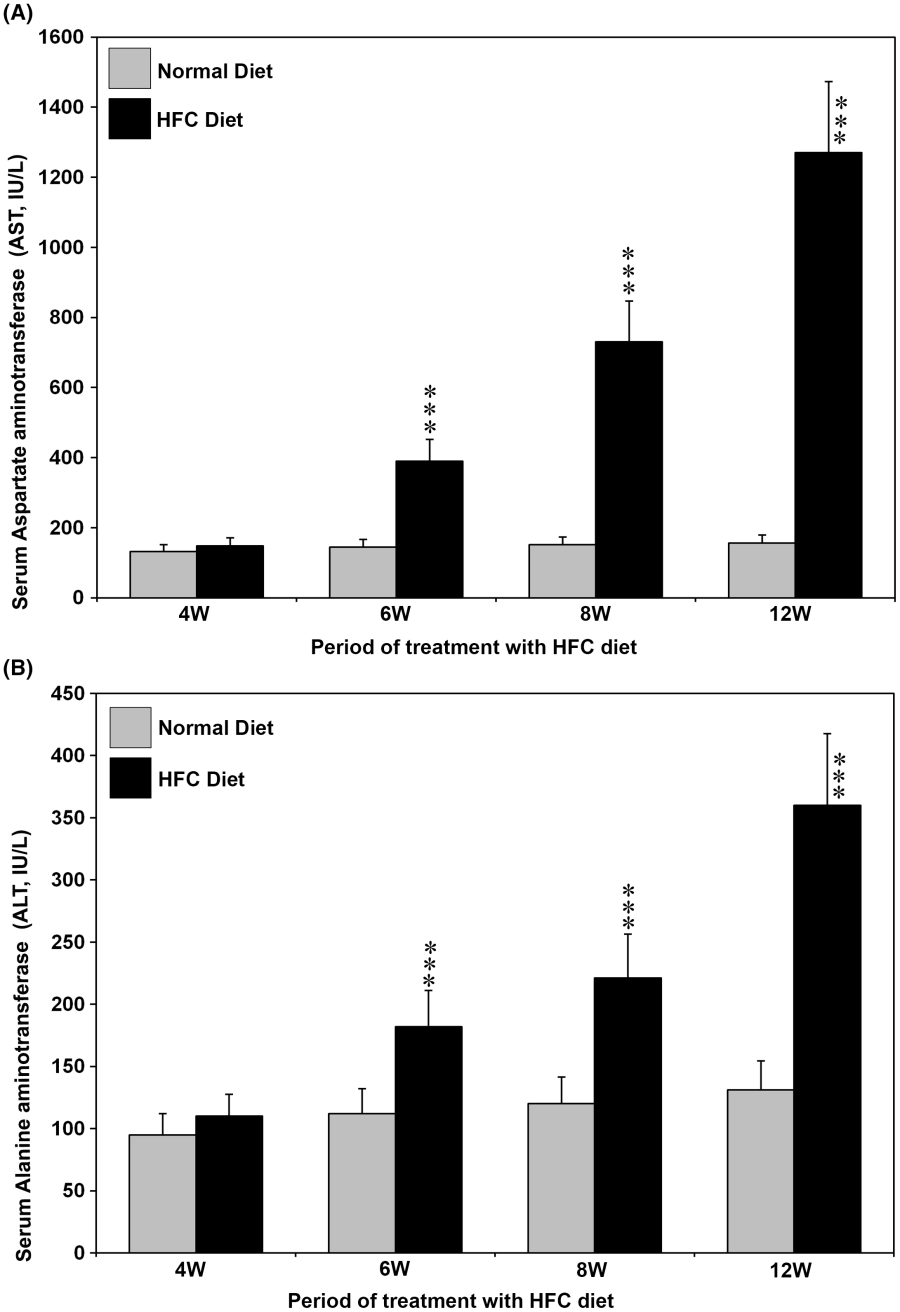
(A) Aspartate aminotransferase (AST) and (B) alanine aminotransferase (ALT) levels in the serum of SHRSP5/Dmcr rats fed with the HFC diet for 12 weeks. Both AST and ALT levels were significantly increased at 6, 8 and 12 weeks in the HFC diet group compared with the animals fed a normal diet. The data are the mean ± SD of five rats in each group. **p < 0.01 and ***p < 0.001 by ANOVA.

The serum levels of AST and ALT during the pathogenesis of MASH and hepatic fibrosis in SHRSP5/Dmcr rats fed a HFC diet are presented in Figure 2. Both AST and ALT levels were significantly increased at 6, 8 and 12 weeks in HFC diet‐fed rats compared with rats fed a normal diet (^*#*^ *p* < 0.001). There was no difference in AST and ALT levels between the rats fed with the HFC diet and normal diet at 4 W.

### 3.3 Oxidative stress and antioxidant status in the hepatic tissue of rats fed with the HFC diet

**FIGURE 3:**
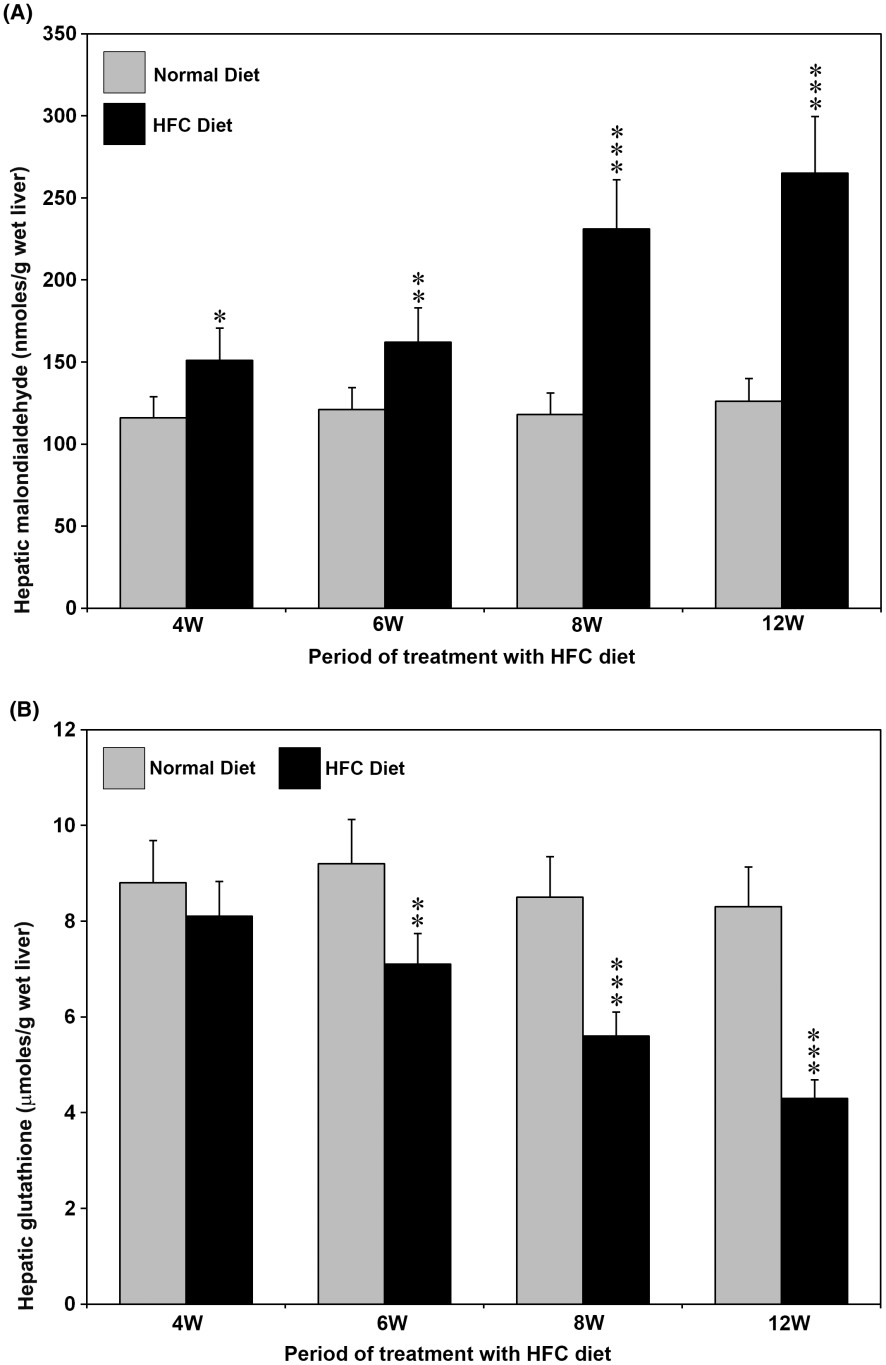
Hepatic malondialdehyde (MDA) and reduced glutathione (GSH) levels in SHRSP5/Dmcr rats fed the HFC diet for 12 weeks. (A) The hepatic MDA levels were markedly increased in the animals fed the HFC diet in all the weeks studied. (B) Hepatic glutathione levels were significantly decreased at 6, 8 and 12 weeks compared with the animals fed a normal diet. The data are the mean ± SD of five rats in each group. *p < 0.05, **p < 0.01 and ***p < 0.001 by ANOVA.

An increased level of malondialdehyde (MDA) is a potent marker of cellular oxidative stress and the peroxidation of membrane lipids.^19^ The hepatic MDA levels in SHRSP5/Dmcr rats fed with the HFC diet are presented in Figure 3A. MDA levels were significantly increased in the liver tissue of animals fed with the HFC diet at all time points studied, indicating increased production of reactive oxygen species (ROS) and elevated oxidative stress compared with the animals fed a normal diet.

Decreased levels of hepatic glutathione (GSH) indicate deterioration of the antioxidant status of the liver. Hepatic GSH levels measured during the administration of the HFC diet in SHRSP5/Dmcr rats are presented in Figure 3B. The mean hepatic GSH levels in HFC diet‐fed animals were significantly reduced at 6, 8 and 12 weeks compared with the animals fed a normal diet at respective time points, indicating decreased antioxidant status. The mean GSH levels at 4 W were not different between the animals fed a normal diet and HFC diet (Figure 3B).

### 3.4 Histopathological assessment of the pathogenesis of MASH and hepatic fibrosis

**FIGURE 4:**
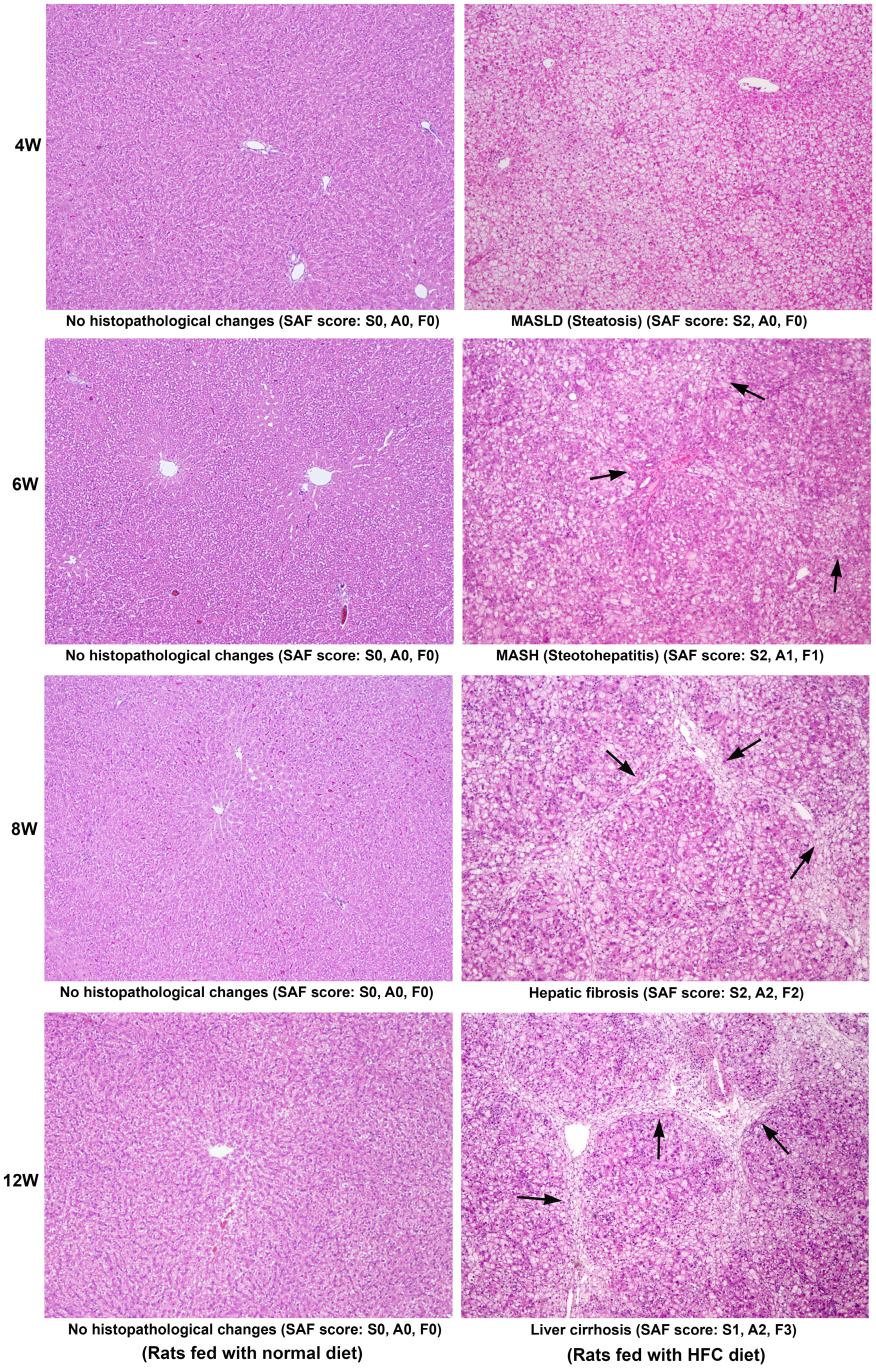
Haematoxylin and Eosin staining in SHRSP5/Dmcr rats fed with a normal and HFC diet for 12 weeks. There was no histopathological alteration in animals fed a normal diet during the entire course of the study. At the 4th week of HFC diet, simple steatosis was present in the entire hepatic parenchyma, indicating the deposition of fat globules. Hepatic fibrosis and inflammation were absent. At the 6th week of HFC diet, fatty degeneration, mild hepatic inflammation and ballooning of hepatocytes were present, especially in the periportal areas (arrows). Moderate periportal fibrosis was present. At the 8th week of HFC diet, hepatic necrosis, sinusoidal congestion and vacuolization of hepatocytes were present. Bridging fibrosis and the formation of collagen fibres were prominent (arrows). Moderate hepatic inflammation was also present. At the 12th week of HFC diet, well‐developed fibrosis and early cirrhosis with the deposition of mature collagen fibres were present (arrows). Moderate‐to‐severe hepatic inflammation and ballooning of hepatocytes were also present. All images are original magnification, ×40. The SAF score, steatosis (S0‐3), activity (A0‐3) and fibrosis (F0‐3) are presented below each representative image.

The histopathological assessment of the liver tissue during the pathogenesis of HFC diet‐induced MASH and hepatic fibrosis in SHRSP5/Dmcr rats, along with the respective controls, is presented in Figure 4. There were no histopathological alterations in the liver tissue of the animals fed a normal diet on any of the days evaluated. There was extensive deposition of fat globules in the hepatic parenchyma of the rats fed with the HFC diet at 4 W. Hepatic necrosis and other pathological alterations were absent. At the 6th week of HFC diet, extreme fatty degeneration, mild hepatic inflammation, ballooning of hepatocytes and periportal fibrosis were present, indicating MASH. In the animals fed with HFC diet for 8 weeks, moderate hepatic necrosis, sinusoidal congestion, incomplete nodule formation and vacuolization of hepatocytes were present. Bridging fibrosis and the deposition of collagen fibres were prominent. At the 12th week of HFC diet administration, well‐developed hepatic fibrosis and early cirrhosis with nodule formation were present. Extensive deposition of thick and matured collagen fibres was prominent. The SAF score assessed by a pathologist, who is unaware of the study, is presented as 0–3 grades below the representative image from each group in Figure 4.

### 3.5 Azan trichrome staining for deposition of collagen in the liver

**FIGURE 5:**
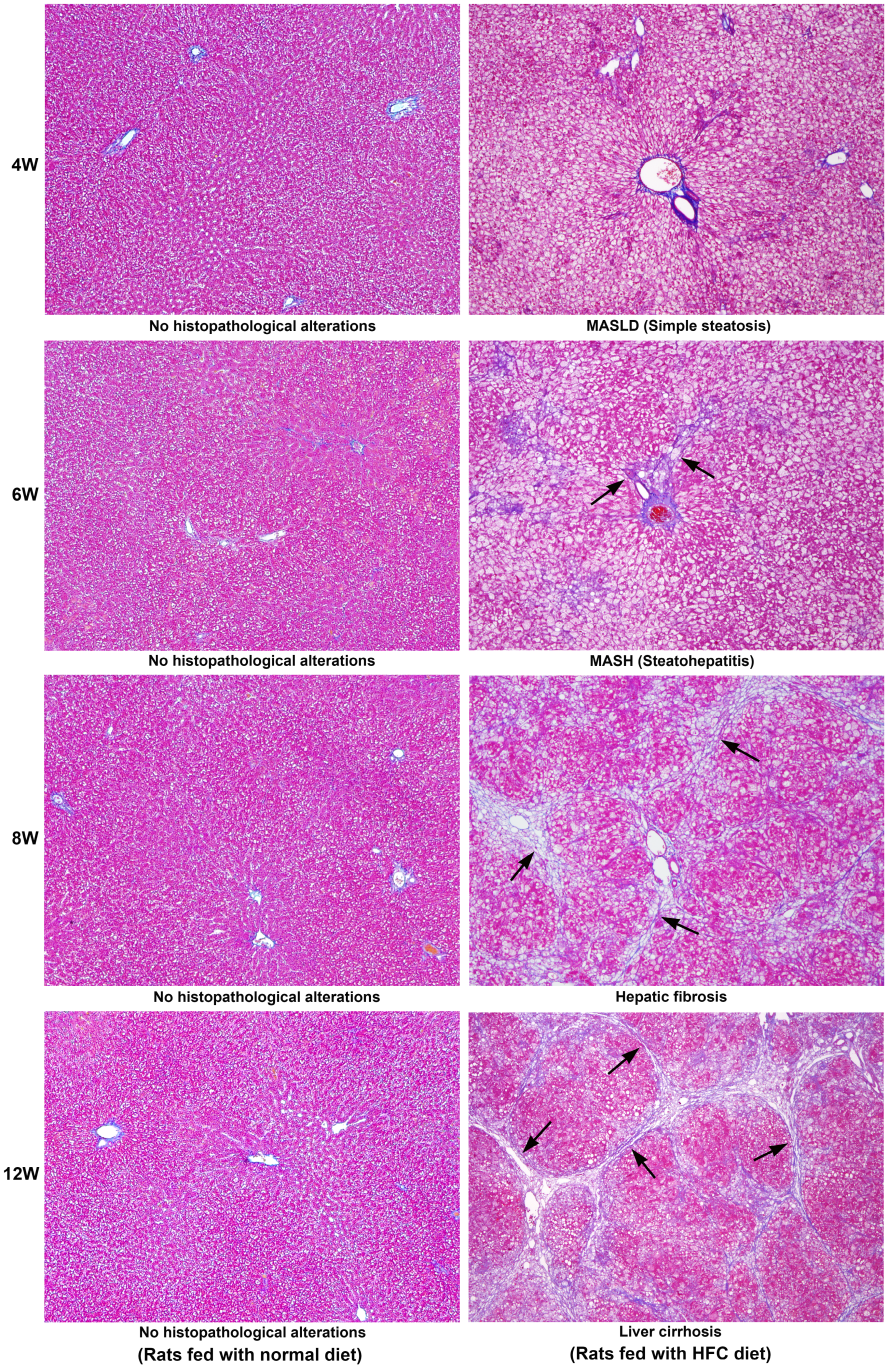
Azan trichrome staining for collagen in SHRSP5/Dmcr rats fed with a normal and HFC diet for 12 weeks. Staining for collagen was completely absent in the hepatic parenchyma of all the animals fed a normal diet. There was no staining for collagen in the liver sections of animals treated with the HFC diet for 4 weeks. At the 6th week of HFC diet, moderate staining of collagen fibres and the initiation of bridging fibrosis were present (arrows). At the 8th week of HFC diet, there was prominent staining for collagen, depicting the formation of thick collagen fibres with well‐developed bridging fibrosis in the hepatic parenchyma (arrows). At the 12th week of HFC diet, marked and intense staining of collagen fibres with well‐developed bridging fibrosis and early cirrhosis was present (arrows). All images are original magnification, ×40.

Azan trichrome staining for collagen in the liver tissue of rats fed the HFC diet for up to 12 weeks is presented in Figure 5. Staining for collagen was absent in the hepatic parenchyma of rats fed a normal diet throughout the study period (Figure 5). There was no staining for collagen fibres in the hepatic parenchyma of animals fed the HFC diet for 4 weeks. However, there were prominent radiating cords from the portal vein with slight staining of thin collagen fibres. At the 6th week of HFC diet, there was moderate staining of collagen fibres in the hepatic parenchyma and the initiation of bridging fibrosis. In rats fed with HFC diet for 8 weeks, there was marked staining for collagen, depicting thick collagen fibres and well‐developed bridging fibrosis in the hepatic parenchyma. Prominent pericentral fibrosis with the formation of fibrous septa was also present. At the 12th week of HFC diet, intense and remarkable staining of thick collagen fibres demonstrating well‐developed hepatic fibrosis and early cirrhosis was prominent (Figure 5). There was conspicuous bridging with early nodule formation, indicating progression towards cirrhosis.

### 3.6 Administration of HFC diet activated hepatic stellate cells

**FIGURE 6:**
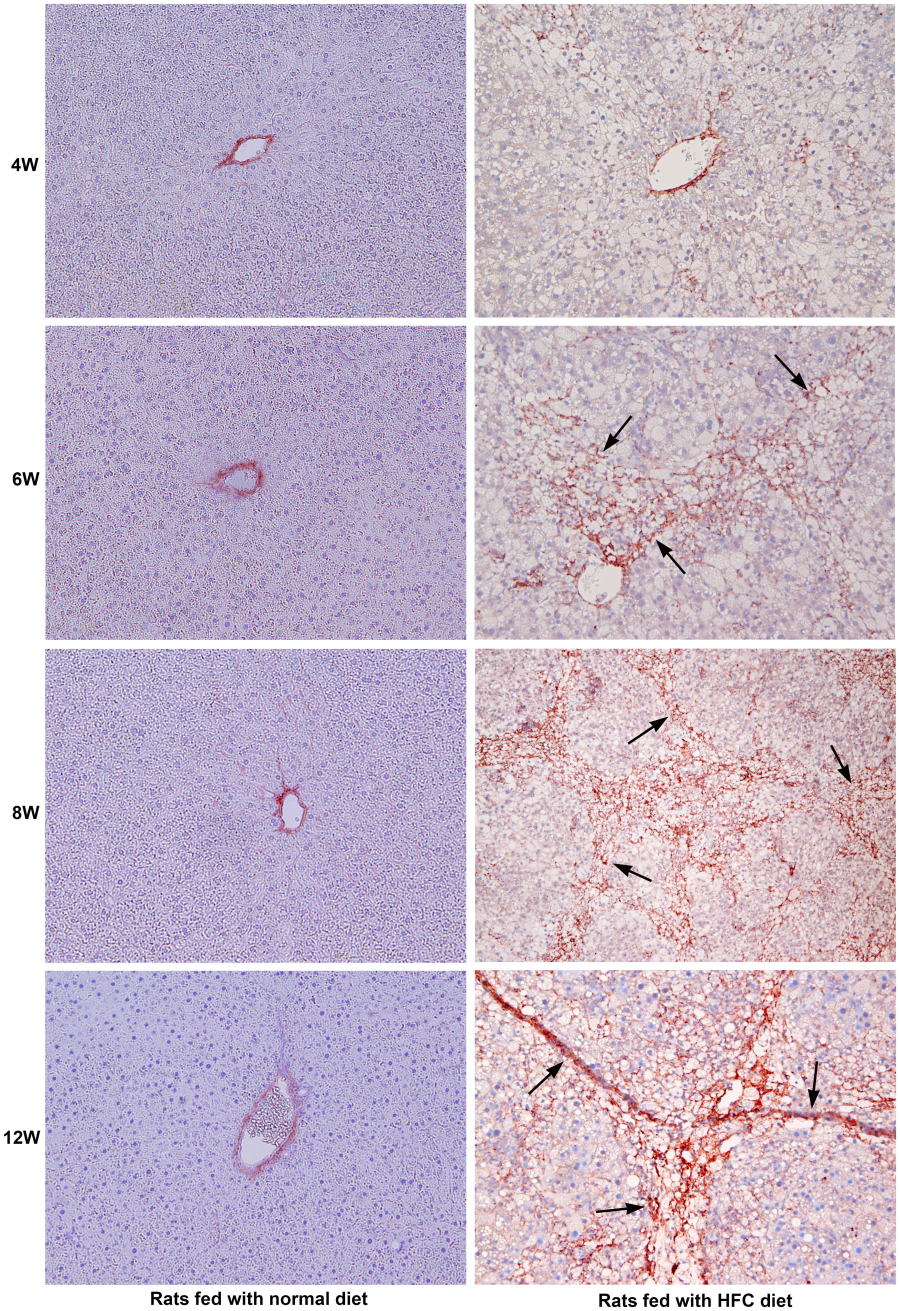
Immunohistochemical staining for α‐smooth muscle actin (α‐SMA) in SHRSP5/Dmcr rats fed with normal and HFC diet for 12 weeks. Staining for α‐SMA was completely absent in the hepatic parenchyma of animals fed a normal diet on all the days studied. Isolated mild staining for α‐SMA was present in the liver sections of rats fed with the HFC diet at the 4th week. At the 6th week of HFC diet, prominent staining for α‐SMA was present in the fibrotic zones, especially in the areas with the initiation of bridging fibrosis (arrows). At the 8th week of HFC diet, enormous and fabulous staining of α‐SMA was present, particularly in the areas with fibrosis (arrows). At the 12th week of HFC diet, strong and remarkable staining for α‐SMA was present in the fibrotic areas, indicating extensive activation of hepatic stellate cells (arrows). All images are original magnification, ×100.

Activation and transformation of resting hepatic stellate cells into myofibroblast‐like cells with the expression of α‐SMA marks the initiation of hepatic fibrosis.^20^ The results of the immunohistochemical staining for the expression of α‐SMA, indicating the activation and transformation of hepatic stellate cells in paraffin liver sections of rats fed with the HFC diet, are presented in Figure 6. Staining for α‐SMA was completely absent in the hepatic parenchyma of SHRSP5/Dmcr rats fed a normal diet throughout the study period (Figure 6). Feeble intermittent staining for α‐SMA was present in the hepatic parenchyma of rats fed with the HFC diet at 4 weeks, indicating the start of activation of hepatic stellate cells. At the 6th week of the HFC diet, there was prominent staining for α‐SMA in the fibrotic zones, especially in the areas with bridging fibrosis. At the 8th week, the rat livers fed with the HFC diet demonstrated prominent and marked staining for α‐SMA, especially in fibrotic zones, indicating the activation of enormous number of hepatic stellate cells. At the 12th week of the HFC diet, there was striking and remarkable staining for α‐SMA in the fibrotic areas, demonstrating extensive activation of hepatic stellate cells (Figure 6).

### 3.7 Treatment with the HFC diet upregulates Collagen Type I expression

**FIGURE 7:**
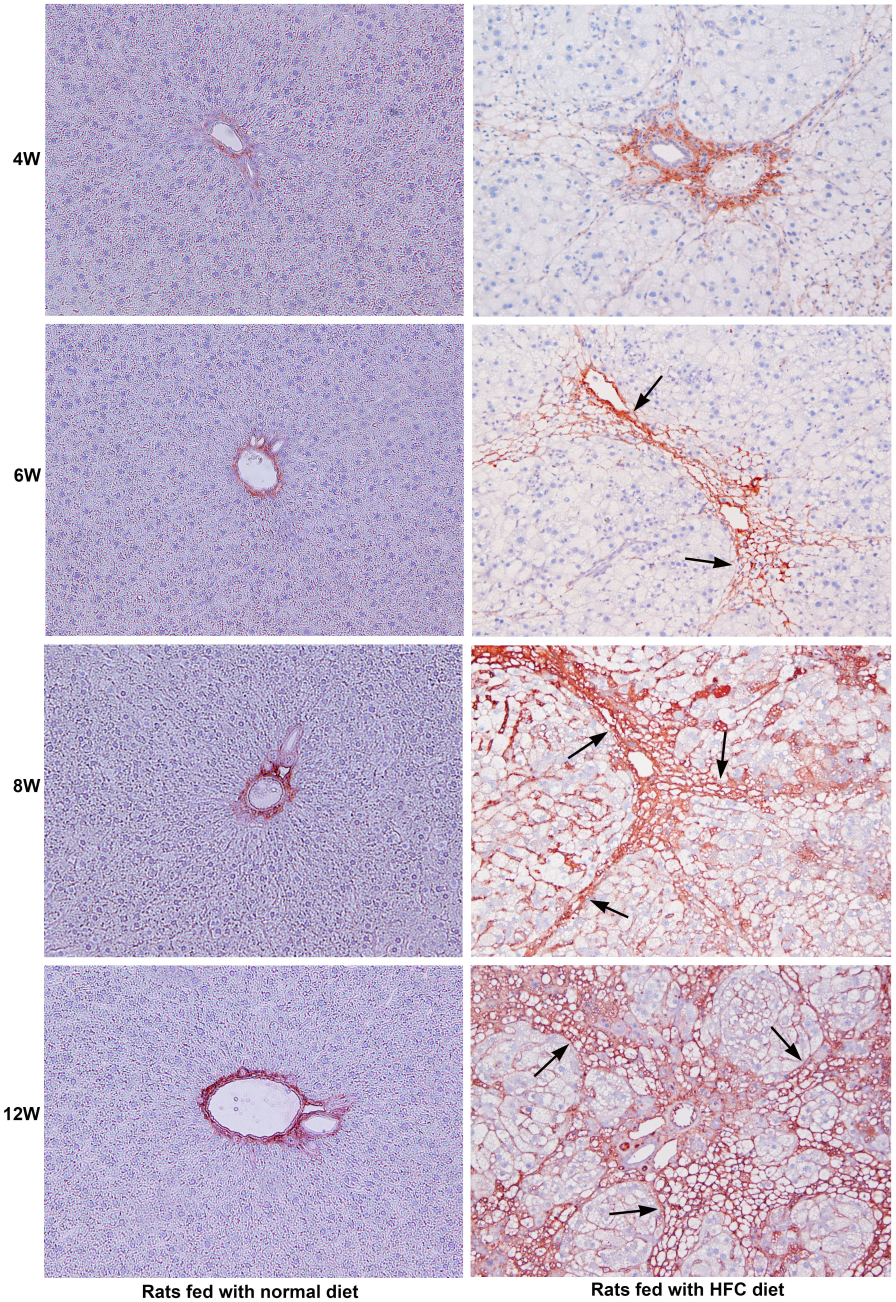
Immunohistochemical staining for Collagen Type I in SHRSP5/Dmcr rats fed a normal and HFC diet for 12 weeks. Staining for Collagen Type I was absent in the hepatic parenchyma of animals fed a normal diet during the entire course of the study. However, distinctive staining for collagen was present in the portal and central veins. Moderate staining for Collagen Type I was present in the hepatic parenchyma as well as in pericentral areas in the livers of animals fed with the HFC diet at the 4th week. At the 6th week of HFC diet, distinct staining for Collagen Type I was present in the fibrotic areas (arrows). At the 8th week of HFC diet, marked and conspicuous staining for Collagen Type I was present in the fibrotic zone with mature collagen fibres (arrows). At the 12th week of HFC diet, strong and remarkable staining for Collagen Type I was present in the fibrotic areas, indicating the deposition of enormous amount of newly formed collagen (arrows). All images are original magnification, ×100.

The deposition of fibril forming collagens in the extracellular matrix of the liver is one of the most prominent features of hepatic fibrosis.^21^, ^22^ The results of the immunohistochemical staining of Collagen Type I in the liver tissue of SHRSP5/Dmcr rats fed the HFC diet are depicted in Figure 7. Staining for Collagen Type I was completely absent in the hepatic parenchyma of rats fed a normal diet during the entire period of the study (Figure 7). Since collagen is present in blood vessels, the hepatic portal and central veins were stained positive for collagen. At the 4th week of the HFC diet, there was moderate staining for Collagen Type I in the periportal areas and mild staining of thin collagen fibres in the hepatic parenchyma. At the 6th week of the HFC diet, moderate and distinct staining of collagen type I was present in the fibrotic areas. At the 8th week of the HFC diet, marked and intense staining for Collagen Type I was present in the fibrotic zone with the deposition of mature collagen fibres. At the 12th week, remarkable and prominent staining of Collagen Type I was present in the fibrotic areas, indicating the deposition of enormous amount of newly formed collagen. The staining of collagen in the liver tissue clearly depicted early nodular cirrhosis.

## 4 DISCUSSION

**FIGURE 8:**
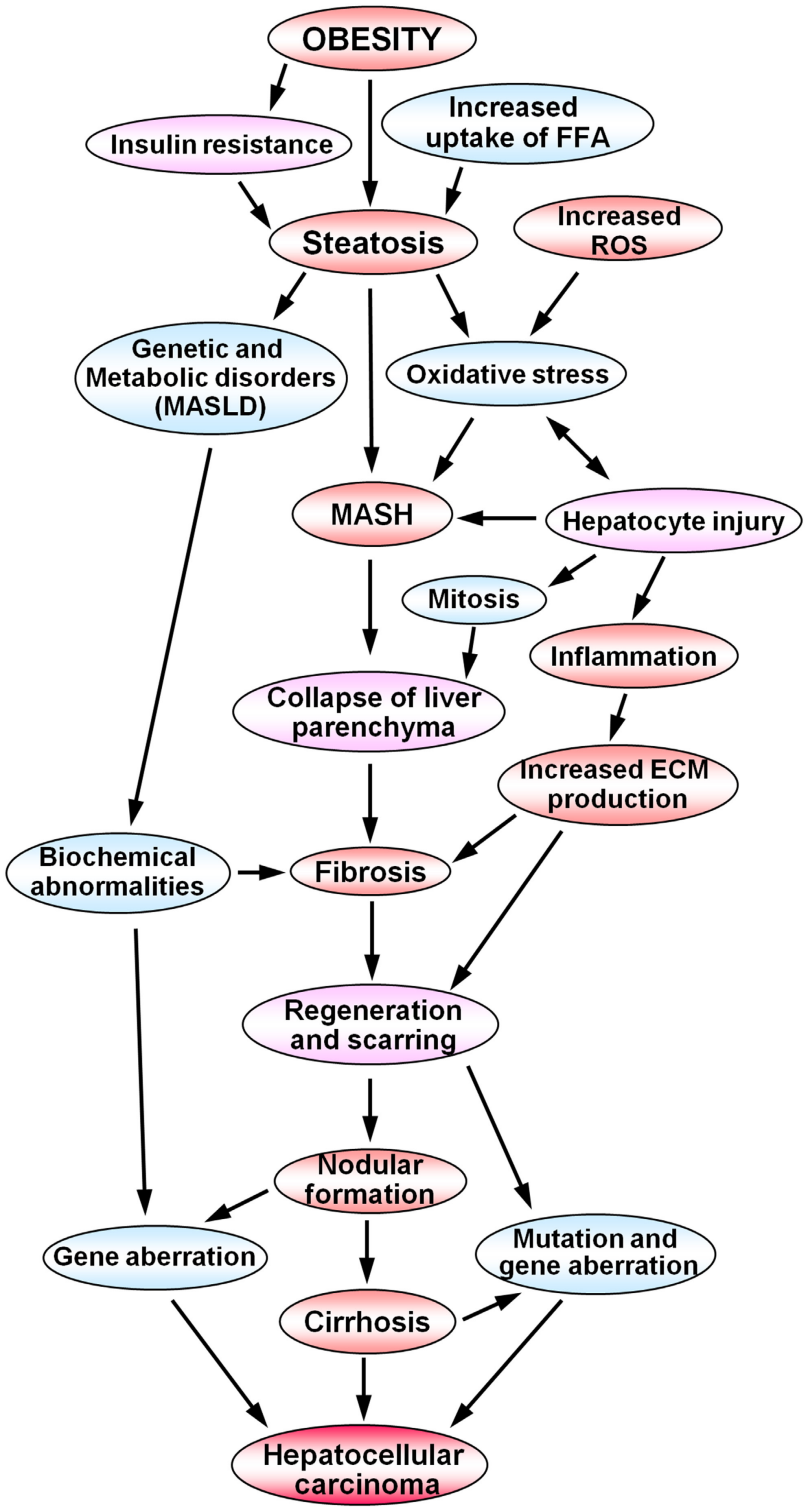
Schematic presentation of the spectrum of metabolic dysfunction‐associated steatotic liver disease depicts the progression of steatosis towards MASH, hepatic fibrosis, liver cirrhosis, and hepatocellular carcinoma. Obesity leads to the accumulation of fat globules in the hepatic parenchyma, which results in increased production of reactive oxygen species and oxidative stress. Uncontrolled and chronic steatosis leads to steatohepatitis, inflammation and hepatocyte injury. These processes induce activation of hepatic stellate cells and increased synthesis and deposition of connective tissue components in the extracellular matrix of the liver, leading to fibrosis. Chronic liver injury and fibrosis lead to nodular formation and liver cirrhosis, which could develop into hepatocellular carcinoma.

Metabolic dysfunction‐associated steatotic liver disease (MASLD) is characterized by increased uptake and deposition of lipid droplets in hepatocytes. Uncontrolled MASLD could progress to MASH, which is accompanied by inflammation, hepatocellular injury and fibrosis that might further transform into liver cirrhosis and hepatocellular carcinoma.^23^ A schematic presentation of the spectrum of MASLD, which depicts the progression of steatosis towards MASH, hepatic fibrosis, liver cirrhosis and hepatocellular carcinoma, is delineated in Figure 8. In the present study, we developed a high‐fat and high‐cholesterol (HFC) diet‐induced rat model of MASH that spontaneously progresses to hepatic fibrosis and liver cirrhosis on continuous feeding of the HFC diet. It is a proper animal model with all the associated biochemical, histopathological and decompensating features of human MASH. It is an easily reproducible and potentially valuable model for studying the mechanisms of the pathogenesis of human MASH, hepatic fibrosis and cirrhosis without employing a chemical agent such as carbon tetrachloride or *N*‐nitrosodimethylamine.^24^, ^25^ Overall, the HFC diet‐induced model of MASH, hepatic fibrosis and liver cirrhosis in SHRSP5/Dmcr rats is an appropriate and suitable animal model to study multiple parameters of human pathological conditions in a natural way.

The sequential elevation of serum AST and ALT is an indication of liver injury and hepatic necrosis, or steatosis.^26^ In the present study, administration of the HFC diet resulted in a consecutive increase in serum AST and ALT levels, indicating hepatocyte injury. Increased oxidative stress and marked decreases in the levels of antioxidants are common features during the pathogenesis of MASH and the accompanied hepatic fibrosis.^27^ The generation of free radicals, including a variety of ROS, leading to cellular oxidative stress and subsequent lipid peroxidation are characteristic features of MASH.^27^ An increased level of serum MDA is an indication of elevated oxidative stress and membrane lipid peroxidation. Malondialdehyde is a final product of the lipid peroxidation of polyunsaturated fatty acids present in cell membranes and one of the best markers of cellular oxidative stress.^28^ In the present study, we observed a marked and sequential increase in hepatic MDA levels during the administration of the HFC diet in SHRSP5/Dmcr rats. On the other hand, a marked decrease in potent antioxidants is also a distinctive feature of MASH and the accompanied hepatic fibrosis. Significantly lower levels of glutathione were reported in a high‐fat, high‐fructose diet‐induced model of non‐alcoholic steatohepatitis.^29^ We have observed a remarkable decrease in the concentrations of the potent antioxidant ascorbic acid during the pathogenesis of N‐nitrosodimethylamine‐induced hepatic fibrosis in rats.^30^ In the current study, hepatic glutathione levels were significantly reduced at 6, 8 and 12 weeks after the start of HFC diet administration, indicating depletion of antioxidant status during the pathogenesis of MASH in SHRSP5/Dmcr rats.

The algorithm for histopathologic classification of liver lesions that covers the entire spectrum of NAFLD (MASLD) is a very appropriate method for scoring the obesity‐associated metabolic syndrome.^16^ In the present study, blind scoring by an experienced pathologist reported activity score, which includes hepatocellular ballooning and lobular inflammation as A2 and the fibrosis score as F2, on a scale of 0–3 at the 8 W of HFC diet administration. The similar scoring was A2 and F3, respectively, at 12 W, which indicates that the current HFC diet‐induced model of MASH is an easily reproducible model with all the decompensating features of human conditions in a natural way within a short period of time without employing any toxic or chemical agent. The control animals fed a normal diet did not depict any histopathological changes during the entire period of the study, which indicates that ageing does not cause any pathological changes in the liver tissue of SHRSP5/Dmcr rats, and all the changes observed in the experimental rats are exclusively from the HFC diet. The Azan trichrome staining for total collagen clearly depicted well‐developed bridging fibrosis and the deposition of mature and thick collagen fibres with nodular formation, which are characteristic features of the initiation of cirrhosis. All these features provide evidence that the current model is highly appropriate and suitable for studying the molecular mechanisms involved in the pathogenesis of MASH and screening potent therapeutic agents to arrest or regress MASLD in human beings.

The hepatic stellate cells (HSCs) are multifunctional nonparenchymal cells located in the space of Disse and are the most prominent cell type involved in the pathogenesis and progression of hepatic fibrosis.^31^ The activation of quiescent hepatic stellate cells is a dynamic process accompanied with the production of various signalling molecules, cytokines and growth factors.^32^ Furthermore, the rate of expression of various cytokines and signalling molecules is a major rate‐limiting event in the degree and progression of hepatic fibrosis.^33^, ^34^ In the present study, immunohistochemical staining demonstrated enormous and remarkable amount of stellate cells in the hepatic parenchyma of rats fed with the HFC diet at 6, 8 and 12 weeks, indicating increased generation and activation of stellate cells. The extent and degree of the staining of activated HSCs coincides with the synthesis and deposition of collagens in the extracellular matrix of the liver.^35^ The staining pattern of HSCs and the increased synthesis and deposition of collagens in the hepatic parenchyma demonstrate the current animal model as a diet‐induced natural model of hepatic fibrosis and cirrhosis.

Excessive synthesis and deposition of fibril‐forming collagens in the extracellular matrix of the liver is the characteristic feature of hepatic fibrosis and liver cirrhosis.^21^, ^36^ We have observed that during the early stages of the pathogenesis and progression of hepatic fibrosis, the rate of synthesis and deposition of Collagen Type III is more prominent than that of Collagen Type I and fibrotic liver collagen is more cross‐linked than normal liver collagen.^37^ However, it was reported that in advanced stages of diet‐induced NASH (the new term MASH), hepatic fibrosis and early cirrhosis, predominantly Collagen Type I deposits in the hepatic parenchyma.^38^ In the present study, immunohistochemical staining for Collagen Type I demonstrated remarkable and extensive staining in the hepatic parenchyma. Furthermore, the staining clearly depicted MASH, hepatic fibrosis and early nodular cirrhosis at the 6th, 8th and 12th weeks of the HFC diet administration, respectively. This clearly indicates that the present model is appropriate to study the molecular events associated with the deposition of collagen in the hepatic parenchyma during the pathogenesis of bridging fibrosis and nodular cirrhosis.

The unique aspect of the current animal model is that here we clearly demonstrated the four stages of metabolic dysfunction‐associated liver disorders, viz., MASLD (simple steatosis) at the 4th week, MASH (steatohepatitis) with progressive fibrosis at the 6th week, well‐developed hepatic fibrosis with bridging at the 8th week, and finally, well‐formed nodular cirrhosis at the 12th week. We have also shown that extensive production of ROS and subsequent cellular oxidative stress contribute to the pathogenesis of the spectrum of MASLD during the sequential administration of HFC diet. In patients with metabolic syndrome, deposition of fat globules in the hepatic parenchyma (simple steatosis) is the first step. Only about 30% of the patients with simple steatosis develop steatohepatitis. Next, about 20% of the patients with steatohepatitis progress to bridging fibrosis and then to nodular cirrhosis.^39^ Therefore, it is important to have an animal model that clearly depicts all four stages of the spectrum of MASLD. Overall, the present study and the associated data demonstrated that the HFC diet‐induced model of steatosis, MASH, hepatic fibrosis and cirrhosis is a feasible, quick and appropriate animal model to study the molecular events associated with the pathogenesis of the spectrum of MASLD and to screen potent therapeutic agents.

## 5 CONCLUSION

The data of the present study clearly demonstrated that the HFC diet‐induced model of MASH, hepatic fibrosis and cirrhosis in rats is an easy, feasible and reproducible animal model for studying the molecular mechanisms involved in the pathogenesis of the spectrum of MASLD and also an appropriate model to screen potent therapeutic agents.

## AUTHOR CONTRIBUTIONS

**Takashi Saito:** Conceptualization (equal); formal analysis (lead); investigation (equal); methodology (lead); resources (supporting); visualization (lead). **Mutsumi Tsuchishima:** Conceptualization (equal); methodology (equal); project administration (lead); resources (equal); supervision (lead). **Mikihiro Tsutsumi:** Funding acquisition (lead); project administration (lead); resources (equal); supervision (supporting); writing – original draft (supporting). **Joseph George:** Data curation (lead); formal analysis (equal); investigation (equal); methodology (lead); writing – review and editing (lead).

## FUNDING INFORMATION

This work was supported by the grant for Research Project from Kanazawa Medical University (Grant #RP 2020‐03) to M. Tsutsumi.

## CONFLICT OF INTEREST STATEMENT

The authors do not have any conflicts of interest to declare in connection with this manuscript.

## Data Availability

The data will be available for verification upon request.
